# Association Between Complete Blood Count Components and Intrahepatic Cholestasis of Pregnancy

**DOI:** 10.7759/cureus.12381

**Published:** 2020-12-30

**Authors:** Jessica Silva, Melissa Magenta, Giovanni Sisti, Lisa Serventi, Kecia Gaither

**Affiliations:** 1 Department of Obstetrics and Gynecology, New York Health and Hospitals/Lincoln, Bronx, USA

**Keywords:** complete blood count, intrahepatic cholestasis of pregnancy, mean platelet volume, neutrophil to lymphocyte ratio, platelet to lymphocyte ratio

## Abstract

Objective

Some components of the routine complete blood count (CBC) and their ratios, such as neutrophil-to-lymphocyte ratio (NLR) and platelet-to-lymphocyte ratio (PLR) have been found to be sensitive biomarkers of preeclampsia and other inflammatory obstetric conditions. We wanted to evaluate whether they can be associated with intrahepatic cholestasis of pregnancy (ICP).

Materials and Methods

We conducted a retrospective case-control study between May 1, 2015 and July 1, 2018. Cases were considered pregnancies with ICP and control healthy pregnancies. Cases and controls were matched for age, parity, and race.

We compared the levels of white blood cells (WBC), hemoglobin, neutrophils, lymphocytes, NLR, PLR, platelets, red cell distribution width (RDW), and mean platelet volume (MPV) in the first and third trimesters between cases and controls. In addition, we compared the same components in the third trimester between patients with mild (serum total bile acid (TBA) of 10 - 40 µmols/L) and severe (TBA > 40 µmols/L) ICP.

Results

There were 33 patients with ICP and 33 controls. There were no significant differences between the two groups in the first trimester. WBC, neutrophil count, and NLR were decreased in women with ICP in the third trimester compared to controls. MPV was significantly higher in the third trimester of patients with ICP compared to controls. RDW was lower in mild ICP compared to severe ICP in the third trimester.

Conclusion

Decreased WBC, neutrophil, NLR, and MPV values are associated with ICP and may be useful additions to the diagnostic algorithm for ICP. Larger studies are needed to assess the responsible underlying molecular pathogenic mechanisms.

## Introduction

Intrahepatic cholestasis of pregnancy (ICP) is generally diagnosed in the late second or third trimester by localized pruritis to the abdomen and palms, elevated serum total bile acids (TBA) (> 10 µmols/L), and abnormal liver function tests, in the absence of other hepatobiliary disorders [1-3].

ICP is the most common hepatobiliary disorder that occurs during pregnancy; the incidence of this disorder appears to vary in accordance with geographic location, time of year, and ethnicity [2, 4-5]. South America is noted to have the highest incidence of ICP (1.5% - 4%), whereas the lowest is seen in Europe (1%) [2]. It is also seen more in the winter months in countries, such as Finland, Sweden, Chile, and Portugal [2]. The exact pathophysiology of ICP is unknown. It is thought to be multifactorial, inclusive of genetic, environmental, nutritional, and endocrinological factors [5]. Studies have shown that ICP might be caused by an impairment in the bile acid transporter and receptor, resulting in bile acids that accumulate in the serum and liver [5]. This increase in bile acids is thought to activate an inflammatory response, leading to damage to the hepatocytes [6-7].

ICP is reversible and tends to spontaneously resolve after delivery. ICP has been linked to premature delivery, meconium passage, fetal oxygen deprivation, and intrauterine fetal death [4, 8].

The current diagnostic gold standard is reached with the occurrence of specific symptoms and laboratory values in the absence of other hepatobiliary disorders in the late second or third trimester [3-4]. The combination of pruritus and biochemical evidence of liver dysfunction, such as elevated serum total bile acid (TBA) (> 10 µmols/L) and abnormal transaminases, lead to the diagnosis [3-4].

An elevation of serum TBA concentration is the most sensitive marker for ICP [3]. Unfortunately, the TBA results are commonly available, on average, 14 days after the serum collection. Clinicians, therefore, require novel laboratory markers of the disease which are readily available to facilitate an earlier diagnosis and prevent adverse perinatal outcomes.

Recent literature has shown that some components of the routine complete blood cell (CBC) count, such as white blood cell count (WBC), hemoglobin, neutrophil count, lymphocyte count, platelet count, red cell distribution width (RDW), mean platelet volume (MPV), and some of their ratios, such as neutrophil to lymphocyte ratio (NLR) and platelet to lymphocyte ratio (PLR), can aid in the diagnosis of malignancies, cardiovascular disease, autoimmune diseases, and some pregnancy pathologies [9-12].

The association of CBC values and ICP has been poorly studied in the current literature. We found only two studies correlating CBC components and pregnancy. In a Turkish study from 2017, Yayla Abide et al. found that MPV was increased in severe ICP cases compared to mild ICP or the control group in the third trimester [13]. In addition, they found a lower RDW, higher WBC, MPV, and PLR in patients with ICP compared to controls.

In Turkey, in 2014, Kirbas et al. found higher NLR levels in the mild ICP group compared to normal pregnancies, and higher NLR levels in the severe ICP group compared to normal pregnancies and mild ICP [14].

We wanted to expand the previous research on the association between CBC components and ICP, including other components of the CBC and their ratios, in the first and third trimester from a population in the United States (US).

## Materials and methods

We conducted a retrospective case-control study at the New York City (NYC) Health and Hospitals/Lincoln, a tertiary care hospital in the Bronx, New York which is part of one of the largest public hospital systems in the US, from May 1, 2015 to July 1, 2018. The Lincoln Medical Center Office of the Institutional Review Board (IRB) approved the study (IRB #19-011). The data was obtained from the hospital's electronic medical records. The IRB waived the consent requirement because the data were collected retrospectively and rendered unidentifiable.

Our primary outcome was to compare the WBC, hemoglobin, neutrophils, lymphocytes, NLR, PLR, RDW, platelets, RDW, and MPV levels in the first and third trimesters between ICP and healthy pregnancies.

Pregnant women with ICP and healthy pregnancies were matched for age, parity, and race.

Diagnostic criteria for ICP included clinical signs/symptoms, such as generalized pruritus without accompanying rash and an elevation of TBA concentration (> 10 µmols/L). Exclusion criteria were patients without a first trimester CBC, patients with preexisting chronic/acute liver and gallbladder diseases, hemolysis, elevated liver enzymes, and low platelet count (HELLP) syndrome, preeclampsia, and other pregnancy-related pathologies.

Our secondary outcome was to compare the levels of WBC, hemoglobin, neutrophils, lymphocytes, NLR, PLR, platelets, RDW, and MPV in the third trimester between patients with mild and severe ICP. Mild or severe ICP classification was based on the level of TBA. A value of 10 - 40 µmols/L was considered mild and > 40 µmols/L was considered severe [2].

Venous blood samples were obtained as part of the routine work-up in the first prenatal visit in the first trimester and upon clinical suspicion of ICP on admission in the third trimester. CBC values were analyzed with our in-house hematology analyzer Beckman Coulter (Beckman Coulter, Inc., Brea, CA, USA). NLR and PLR were calculated as ratios.

The statistical analysis was performed with Statistical Package for Social Sciences (SPSS) v. 22.0 (IBM SPSS Statistics, Armonk, NY). 

The continuous variables were expressed with a median (25°^ ^to 75° percentile) and the categorical variables were indicated by ratio to the total.

We assessed the normality of the continuously distributed variables with the Shapiro-Wilk test: all the data significantly deviated from a normal distribution.

To compare the study groups, we used the non-parametric Mann-Whitney test for continuous variables and the chi-square test for the categorical variables. A p-value of less than 0.05 was considered statistically significant.

## Results

We included 33 patients with ICP and 33 patients with healthy pregnancies. They were matched for age, race, and parity (Table 1). There were no differences in CBC components, NLR, or PLR levels between the two groups in the first trimester (Table 2).

**Table 1:** Demographics of Patients with ICP and Healthy Pregnancies * Chi-square test **Mann-Whitney test ICP: intrahepatic cholestasis of pregnancy; NS: not significant

	ICP	Controls	p-value
Parity > 0/Total	27/33	26/33	NS*
Age (years)	30 (27 - 32)	28 (23 - 32)	NS**
Hispanic/Total	29/33	26/33	NS*

**Table 2:** First Trimester Complete Blood Cell Components in ICP Versus Healthy Pregnancies *Mann-Whitney test ICP: intrahepatic cholestasis of pregnancy; MPV: mean platelet volume; NLR: neutrophil-to-lymphocyte ratio; PLR: platelet-to-lymphocyte ratio; RDW: red cell distribution width; WBC: white blood cells

	ICP	Controls	p-value*
WBC (K/µL)	7.4 (6.8 - 9.2)	8.0 (6.7 - 9.8)	0.30
Hemoglobin (g/dL)	12.6 (12 - 12.9)	12.5 (11.9 - 13.3)	0.68
Neutrophil (K/µL)	5.0 (4.6 - 6.7)	5.3 (4.3 - 6.4)	0.94
Lymphocyte (K/µL)	2.0 (1.3 - 2.4)	1.8 (1.6 - 1.9)	0.73
NLR	2.7 (2.2 - 3.3)	2.9 (2.1 - 3.7)	0.56
PLR	121.7 (94.2 - 177.7)	147.8 (130.1 - 173.5)	0.22
Platelet (K/µL)	232 (210 - 270)	277 (220 - 309)	0.15
RDW (%)	13.6 (13.2 - 14.1)	13.8 (13.1 - 13.9)	0.86
MPV (fL)	9.2 (8.6 - 10)	9.0 (8.3 - 9.8)	0.38

A significant increase in MPV was noted in patients with ICP compared to healthy controls (9.8 fL vs 9.1 fL, p < 0.01) in the third trimester. WBC (7.7 K/uL vs 10.5 K/uL, p < 0.01), neutrophil count (5.4 K/uL vs 8 K/uL, p < 0.01), and NLR (3.2 vs 4.2, p < 0.01) were found to be decreased in ICP compared to healthy controls in the third trimester (Table 3). 

**Table 3:** Third Trimester Complete Blood Cell Components in ICP Versus Healthy Pregnancies *Mann-Whitney test ICP: intrahepatic cholestasis of pregnancy; MPV: mean platelet volume; NLR: neutrophil-to-lymphocyte ratio; PLR: platelet-to-lymphocyte ratio; RDW: red cell distribution width; WBC: white blood cells

	ICP	Controls	p-value*
WBC (K/µL)	7.7 (6.7 - 8.9)	10.5 (9.2 - 11.8)	< 0.01
Hemoglobin (g/dL)	12.0 (11.3 - 12.8)	12.3 (12.0 - 13.0)	0.40
Neutrophil (K/µL)	5.4 (4.0 - 6.4)	8.0 (5.6 - 9.4)	< 0.01
Lymphocyte (K/µL)	1.7 (1.4 - 1.9)	1.8 (1.4 - 2.4)	0.62
NLR	3.2 (2.5 - 3.7)	4.2 (2.5 - 6.4)	< 0.01
PLR	122.3 (85.2 - 157.9)	120 (99.6 - 157.6)	0.47
Platelet (K/µL)	213.0 (161.0 - 239.0)	228 (183.0 - 268.0)	0.09
RDW (%)	14.3 (13.4 - 15.6)	14.0 (13.5 - 14.8)	0.38
MPV (fL)	9.8 (9.1 - 10.9)	9.1 (8.4 - 9.8)	< 0.01

RDW was lower in mild ICP compared to severe ICP cases (13.3 vs 14.05, p = 0.014) in the third trimester (Table 4).

**Table 4:** Third Trimester Complete Blood Cell Components in Mild Versus Severe Cases of ICP *Mann-Whitney test ICP: intrahepatic cholestasis of pregnancy; MPV: mean platelet volume; NLR: neutrophil-to-lymphocyte ratio; PLR: platelet-to-lymphocyte ratio; RDW: red cell distribution width; WBC: white blood cells

	Mild ICP (10 - 40 μmol/L)	Severe ICP (> 40 μmol/L)	p-value*
WBC (K/µL)	8.2 (7.1 - 10.1)	7.15 (6.5 - 7.9)	0.22
Hemoglobin (g/dL)	12.8 (12.1 - 13.4)	12.5 (11.9 - 12.9)	0.19
Neutrophil (K/µL)	6.0 (4.7 - 6.9)	4.7 (4.2 - 5.9)	0.41
Lymphocyte (K/µL)	1.7 (1.3 - 2.4)	2.0 (1.4 - 2.4)	0.65
NLR	3.1 (2.3 - 3.8)	2.6 (2.1 - 3.2)	0.32
PLR	145.3 (90.5 - 176.2)	115.0 (99.9 - 178.6)	0.71
Platelet (K/µL)	235.0 (200.5 - 257.5)	230.0 (220.7 - 284.5)	0.65
RDW (%)	13.3 (13.1 - 13.7)	14.05 (13.5 - 14.4)	0.014
MPV (fL)	9.5 (8.8 - 10.4)	9.1 (8.9 - 9.7)	0.42

## Discussion

In our study, MPV was significantly elevated in patients diagnosed with ICP in the third trimester compared to healthy pregnancies. Our result is in accordance with a study done in Turkey by Yayla Abide et al. where they found MPV to be elevated in patients with ICP [13].

MPV can be elevated in clinical conditions associated with an underlying inflammatory process and, as such, can be utilized as a marker denoting the course and prognosis in many medical conditions [12]. MPV, being a marker for platelet activity, changes in response to the number of platelets [12]. In inflammatory pathologic conditions, there is an increased platelet aggregation and an increase in the percentage of larger platelets secondary to the production of inflammatory mediators causing an increase in MPV [12]. As ICP is thought to be caused by inflammation of the hepatocytes from elevated bile acids an increase in MPV is plausible [6].

We saw a significant decrease in NLR in the ICP group compared to the control group in our study. In contrast, there was a significant elevation in NLR in the Kirbas et al. study [14], and in the study by Yayla Abide et al., the NLR levels were similar between both groups [13].

In our study, the RDW was lower in mild ICP compared to severe ICP in the third trimester. This is in contrast with the study by Yayla Abide et al. published in 2017, where they found no association between the RDW and ICP severity [13]. 

The contradicting results might be explained by the limited number of cases in our study.

We saw a significant decrease of WBC and neutrophil count in ICP compared to healthy controls in the third trimester. 

Elevated bile acids cause damage to the hepatocytes, resulting in an inflammatory response. In this response, there is the recruitment of lymphocytes and neutrophils to the liver and out of the bloodstream [6, 11]. Our blood samples in the third trimester were drawn at the time of the first clinical suspicion of ICP. We hypothesize that the decrease in WBC, neutrophils, and NLR in the ICP group was due to a reaction of the immune system to the hepatocyte injury. This injury leads to the recruitment of immune cells from the bloodstream to the liver, thereby decreasing their levels in the sera. Together, this can explain the lower numbers of WBC and neutrophils circulating in the blood.

None of the analyzed components of the CBC were found to be significantly different between ICP and controls in the first trimester.

We speculate that the lack of differences in the first trimester might be related to the underlying pathogenesis of ICP. Indeed, ICP might be caused by specific fetus-placenta-liver interactions in the third trimester, as opposed to other pregnancy-related inflammatory diseases, such as preeclampsia, which has its hallmarks beginning in the early stages of placentation.

This observation additionally is in accordance with previous studies by our research group where we noted specific alterations of the CBC components only in the third trimester for a similar obstetric pathology of the liver during pregnancy, the HELLP syndrome [15-16].

## Conclusions

Larger studies are needed to assess the underlying molecular pathogenic mechanisms and to further explore the trends that where founded in this study. If confirmed by future studies, some of the CBC components and their ratios could be incorporated into a new diagnostic algorithm for ICP.

## References

[1] Chivers S, Williamson C (2018). Intrahepatic cholestasis of pregnancy. Obstet Gynaecol Reprod Med.

[2] Geenes V, Williamson C (2009). Intrahepatic cholestasis of pregnancy. World J Gastroenterol.

[3] Pathak B, Sheibani L, Lee RH (2010). Cholestasis of pregnancy. Obstet Gynecol Clin North Am.

[4] Brouwers L, Koster MP, Page-Christiaens GC (2015). Intrahepatic cholestasis of pregnancy: maternal and fetal outcomes associated with elevated bile acid levels. Am J Obstet Gynecol.

[5] Krishna I, Lindsay M (2015). Intrahepatic cholestasis of pregnancy. Topics Obstet Gynecol.

[6] Allen K, Jaeschke H, Copple BL (2011). Bile acids induce inflammatory genes in hepatocytes: a novel mechanism of inflammation during obstructive cholestasis. Am J Pathol.

[7] Woolbright BL, Jaeschke H (2012). Novel insight into mechanisms of cholestatic liver injury. World J Gastroenterol.

[8] Kawakita T, Parikh LI, Ramsey PS (2015). Predictors of adverse neonatal outcomes in intrahepatic cholestasis of pregnancy. Am J Obstet Gynecol.

[9] Zhang WW, Liu KJ, Hu GL, Liang WJ (2015). Preoperative platelet/lymphocyte ratio is a superior prognostic factor compared to other systemic inflammatory response markers in ovarian cancer patients. Tumour Biol.

[10] Kim NY, Chun DH, Kim SY (2019). Prognostic value of systemic inflammatory indices, NLR, PLR, and MPV, for predicting 1-year survival of patients undergoing cytoreductive surgery with HIPEC. J Clin Med.

[11] Núñez J, Núñez E, Bodí V (2008). Usefulness of the neutrophil to lymphocyte ratio in predicting long-term mortality in ST segment elevation myocardial infarction. Am J Cardiol.

[12] Korniluk A, Koper-Lenkiewicz OM, Kamińska J, Kemona H, Dymicka-Piekarska V (2019). Mean platelet volume (MPV): new perspectives for an old marker in the course and prognosis of inflammatory conditions. Mediators Inflamm.

[13] Yayla Abide Ç, Vural F, Kılıççı Ç, Bostancı Ergen E, Yenidede İ, Eser A, Pekin O (2017). Can we predict severity of intrahepatic cholestasis of pregnancy using inflammatory markers?. Turk J Obstet Gynecol.

[14] Kirbas A, Biberoglu E, Daglar K (2014). Neutrophil-to-lymphocyte ratio as a diagnostic marker of intrahepatic cholestasis of pregnancy. Eur J Obstet Gynecol Reprod Biol.

[15] Sisti G, Faraci A, Silva J, Upadhyay R (2019). Neutrophil-to-lymphocyte ratio, platelet-to-lymphocyte ratio, and complete blood count components in the first trimester do not predict HELLP syndrome. Medicina (Kaunas).

[16] Sisti G, Faraci A, Silva J, Upadhyay R (2019). Neutrophil-to-lymphocyte ratio, platelet-to-lymphocyte ratio, and routine complete blood count components in HELLP syndrome: a matched case control study. Medicina (Kaunas).

